# Multifunctional Cargo-Free Nanomedicine for Cancer Therapy

**DOI:** 10.3390/ijms19102963

**Published:** 2018-09-28

**Authors:** Ying Wang, Pengfei Yang, Xinrui Zhao, Di Gao, Na Sun, Zhongmin Tian, Tianyou Ma, Zhe Yang

**Affiliations:** 1 The Key Laboratory of Biomedical Information Engineering of Ministry of Education, School of Life Science and Technology, Xi’an Jiaotong University, Xi’an 710049, China; wy1127@stu.xjtu.edu.cn (Y.W.); pengfeiyang323@stu.xjtu.edu.cn (P.Y.); zhaoxinrui0103@stu.xjtu.edu.cn (X.Z.); digao5-c@my.cityu.edu.hk (D.G.); shanqilingyun@stu.xjtu.edu.cn (N.S.); zmtian@mail.xjtu.edu.cn (Z.T.); 2 Institute of Endemic Diseases, Environment and Diseases-Related Gene of Key Laboratory of Education Ministry, Medical School of Xi’an Jiaotong University, Xi’an 710061, China; 3 Department of Ophthalmology, University of California, San Diego, La Jolla, CA 92093, USA

**Keywords:** cargo-free, nanomedicine, combination therapy, cancer therapy

## Abstract

Nanocarriers encapsulating multiple chemotherapeutics are a promising strategy to achieve combinational chemotherapy for cancer therapy; however, they generally use exotic new carriers without therapeutic effect, which usually suffer from carrier-related toxicity issues, as well as having to pass extensive clinical trials to be drug excipients before any clinical applications. Cargo-free nanomedicines, which are fabricated by drugs themselves without new excipients and possess nanoscale characteristics to realize favorable pharmacokinetics and intracellular delivery, have been rapidly developed and drawn much attention to cancer treatment. Herein, we discuss recent advances of cargo-free nanomedicines for cancer treatment. After a brief introduction to the major types of carrier-free nanomedicine, some representative applications of these cargo-free nanomedicines are discussed, including combination therapy, immunotherapy, as well as self-monitoring of drug release. More importantly, this review draws a brief conclusion and discusses the future challenges of cargo-free nanomedicines from our perspective.

## 1. Introduction

Cancer is one of the most serious life-threatening diseases [1] and the World Health Organization (WHO) predicted that the number of new cancer cases would increase continuously up to 19.3 million by 2025. Among a series of cancer treatments currently applied in clinical practice, including conventional (surgery, chemotherapy and radiotherapy) and innovative modalities (e.g., phototherapy, immunotherapy and gene therapy), chemotherapy still occupies a crucial position [2,3]. As systemic treatment, chemotherapy can not only shrink the cancer before radiation therapy and surgery but also eliminate metastatic cancer cells due to the widespread body distribution of anticancer drugs in the bloodstream [4,5]. However, there are still many obvious drawbacks to chemotherapy. It has been widely recognized that severe side effects could be caused during treatment due to the nonspecific in vivo distribution of traditional chemotherapeutics. Additionally, the occurrence of multidrug resistance (MDR) after repeated administration of same chemotherapeutic agents could also compromise the chemotherapeutic effect [6]. Thus, it is necessary to develop more effective ways to improve the performance of chemotherapy by fostering its strengths and circumventing its weaknesses.

The delivery of therapeutics using drug carriers appears as a promising and innovative approach to improve cancer treatment. It is largely attributed to the unique benefits of carriers in improving the circulation and therapeutic index of therapeutics and reducing the adverse effects of them on normal tissue [6]. With the development in nanotechnology over past few decades, various nanoformulations have been studied and applied in cancer therapy, including liposomes, micelles, albumin nanoparticles, polymeric nanoparticles and inorganic nanoparticles [7,8]. More importantly, several nanomedicines have been approved for clinical application (Table 1). For example, Doxil^®^ (doxorubicin-loaded PEGylated liposomes) was the first nanomedicine approved by the U.S. Food and Drug Administration (FDA), which is commercially available in the United States. It had shown an enhanced therapeutic effect against acquired immune-deficiency syndrome (AIDS)-related Kaposi’s sarcoma and ovarian cancers than standard therapies [9]. In addition, Abraxane^®^ (an albumin-bound paclitaxel nanoparticle) could even generate $967 million in annual revenue due to its impressive therapeutic efficiency by improving its pharmacokinetic properties of paclitaxel compared to Taxol^®^ [10,11].

Although these carrier-assistant nanomedicine present advantages in combating cancers, there are still some limitations. Firstly, the process of fabricating nanocarriers is extremely complicated in most cases. The challenges that were confronted during materials synthesis and purification, as well as nanoparticle preparation, could prevent their production on a large scale [12,13,14]. Secondly, almost all nanocarriers for therapeutics delivery are used as excipients without any therapeutic effect. For some particular carriers, their degradations can even induce some adverse effects (e.g., mitochondrial damage and cardiovascular effects) and immune reactions [14,15,16]. For instance, although poly(lactic acid) (PLA) as a biomaterial has been approved by FDA, it can induce a local inflammatory response following in vivo application [17,18]. Mesoporous silica nanoparticles (MSNs), an excellent candidate among drug carriers, present high drug-loading capacity due to their large surface area [19,20]. However, the long-term toxicity and ineffective biodegradability of MSNs may limit their further application as a drug carrier [15]. Thus, it is necessary to design and prepare innovative nanomedicine platforms to take advantage of nanoformulation without using any unnecessary material. 

**Figure 1:**
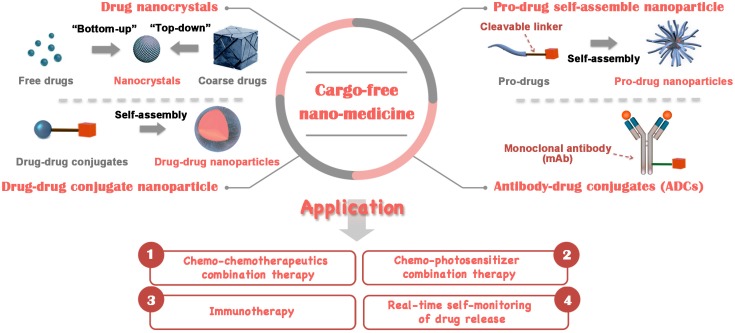
Overview of classification and representative applications of cargo-free nanomedicine.

Cargo-free nanomedicine was formed through self-assembly of therapeutics in the absence of extraneous nanocarriers. This novel nanomedicine has been considered as an excellent paradigm for effective cancer therapy for the following reasons: (1) Cargo-free nanomedicine could prevent drugs from rapid blood/renal clearance by altering their aggregation state. It can also facilitate drugs to accumulate at tumor tissue through the enhanced permeability and retention (EPR) effect due to their reserved nanoscale features; (2) circumvention of additional carrier materials when preparing cargo-free nanomedicine could not only improve drug-loading efficiency (even up to 100% for drug nanocrystals) but also allay concerns about the biosafety of these carrier materials, such as their toxicity and immunogenicity; (3) compared with conventional drug-delivery systems, cargo-free nanomedicine is composed of small molecule units, usually resulting in smaller size and higher penetrability in tumor tissue. These superiorities promote cargo-free nanomedicine to overcome multilayered stromal-cell barriers for deeper tumor penetration and drug perfusion; (4) large-scale fabrication of cargo-free nanomedicine is easily achievable due to their comparatively simple structure [21,22,23,24]. Due to the aforementioned advantages, cargo-free nanomedicine has been considered a promising strategy for future clinical applications. Therefore, the objectives of this review are to present an overview of cargo-free nanomedicine, summarize their classification and describe representative applications of these carrier-free nanomedicines for different purposes, including combinational therapy, immunotherapy and self-monitoring (Figure 1). Moreover, this review presents a brief conclusion and discusses the future challenges of cargo-free nanomedicine from our perspective.

## 2. Classification of Cargo-Free Nanomedicine

Cargo-free nanomedicine refers to drug-delivery nanosystems, with no nontherapeutic ingredients, which are used in cancer treatment. According to the difference in the building blocks of these nanosystems, cargo-free nanomedicine could be classified into drug nanocrystals, prodrug self-assembled nanoparticles, drug–drug conjugate nanoparticles and antibody-drug conjugates (ADCs). Drug nanocrystals are a representative example of a self-delivery system aggregated by pure drugs. These nanocrystals could be formulated by one drug alone or multiple drugs and 100% drug-loading capacity is achievable with them. Compared with nanocrystals that consist of disordered, kinetically trapped chains, self-assembled nanomedicine is in a dynamic equilibrium, showing a high level of internal order as well as enhanced stability. However, the hydrophobicity of anticancer drugs usually limits their self-assembly in aqueous environments. Upon introducing hydrophilic groups into drug molecules, the formation of nanoparticles via self-assembly could easily be achieved. Prodrug self-assembled nanoparticles are a type of nanoparticle assembled from drugs predecorated with small groups via susceptible linkers. Amphiphilic drug–drug conjugates consisting of hydrophobic and hydrophilic anticancer drugs were also designed for self-assembling nanoparticles for drug delivery. ADCs, containing three crucial components, that is, monoclonal antibodies (mAb), cytotoxins and chemical linkers, are also cargo-free nanomedicine, showing potential in enhancing cancer-therapy efficacy. The following sections discuss these types of cargo-free nanomedicine in detail with the assistance of several representative examples.

### 2.1. Drug Nanocrystals

Drug nanocrystals are drug crystals with a nanoscale particle size. They are usually composed of almost 100% hydrophobic drugs and small amounts of ionic or nonionic surfactants to maintain their stability [25,26,27]. Currently, there are three major approaches for fabricating drug nanocrystals, “top–down,” “bottom–up” and combination methods [25,28]. In the “top–down” method, high-pressure homogenization (HPH) [29] or wet-ball milling [30] is adopted to decrease the size of coarse drugs down to nanocrystals; conversely, the “bottom–up” method refers to the self-assembly of drugs into nanoscale particles by nanoprecipitation [31]. Some thermolabile drugs could also experience controlled crystallization during freeze-drying with this method [28,32]. For combination techniques, the “top–down” and “bottom–up” methods are well-integrated with the purpose of fabricating a nanoscale and narrowing size distribution of drug nanocrystals [33,34,35]. So far, a series of nanocrystal formulations have been prepared and studied for cancer therapy by using the aforementioned methods. Excitingly, six therapeutic nanocrystal products have been commercially available since the 1990s and many others are undergoing clinical trials [25,28]. For example, Theralux^®^, which is fabricated by Thymectacin, has been studied in a Phase I/II trial for colon-cancer treatment [28]. Panzem^®^-NCD, a composite of 2-methoxyestradiol nanocrystals (a natural metabolite of estradiol), can efficiently inhibit angiogenesis. For this reason, the combinational treatment of Panzem^®^-NCD and bevacizumab or sunitinib are used to treat metastatic carcinoid tumors in Phase II clinical trials [36].

The fabrication of two drugs into single-drug nanocrystals has also proven to be another promising strategy to achieve enhanced therapy efficiency through the synergistic effect of these drugs. For instance, Liang’s group used a simple reprecipitation method to successfully mix doxorubicin (DOX) and 10-hydroxycamptothecin (HCPT) into a single nanoparticle in the absence of any nanocarrier or solubilizing surfactants [37]. This dual-drug nanocrystal showed enhanced synergistic cytotoxicity to suppress breast cancer. Additionally, Shen’s group prepared an amphiphilic antitumor drug (irinotecan hydrochloride, CPT11) as surfactant to emulsify hydrophobic drugs such as camptothecin (CPT) or paclitaxel (PTX) into nanodispersions [13]. Based on their results, both drug bioavailability and anticancer ability improved significantly by employing cargo-free nanomedicine. More importantly, ready clinic translation is more achievable by avoiding additional carriers.

### 2.2. Prodrug Self-Assembly Nanoparticles

Amphiphilic prodrugs could self-assemble into nanoparticles through intermolecular interaction between their hydrophobic moieties [38,39]. Through using insoluble drugs as these hydrophobic segments, amphiphilic prodrugs with low molecular weight have been successfully synthesized and formulated into a series of cargo-free prodrug self-assembly nanoparticles. Furthermore, due to the presence of some stimuli-responsive cleavable linkers in prodrugs, such as enzyme- [40], acid- [41] and redox-sensitive linkers [42], prodrug nanoparticles can achieve controlled release of the drugs under a special tumor environment or external stimuli [43]. Attributed to overexpressed enzymes/specific secretions, abnormal pH and redox potential in tumor tissue or cells, biologically inactive drugs could readily transform into pharmacologically active ones, leading to the effective inhibition of tumor growth. Chen’s group synthesized the acid-sensitive PEGylated doxorubicin (mPEG-CAD) by conjugation of PEG with *cis*-aconitic anhydride-modified DOX (CAD) [41]. The resultant mPEG-CAD was able to self-assemble into micelles in PBS at pH 7.4, which exhibited the clear spherical morphology with the diameter of 100 nm. Moreover, due to its pH-sensitive release, this prodrug nanoparticle possessed enhanced antitumor efficacy as well as upregulated security in vivo compared to free DOX. 

Matrix metalloproteinases (MMPs) are overexpressed in a tumor extracellular matrix [44]. Thus, activation of prodrugs by MMPs is another smart way to induce the disassembly of prodrug self-assembly nanoparticles and the controlled release of drugs, leading to enhanced therapeutic efficiency [40,45]. Maruyama’s group prepared an MMP7-sensitive precursor of hydrogelation [46]. In the absence of MMP7, the hydrogelation of the precursor solution was inhibited due to the conjugation of the gelator with gelation-preventing moiety. Once taken up by cancer cells, the MMP7-sensitive peptide linkers between these two parts could be cleaved, finally converting into a hydrogel inside the cancer cell. This process resulted in vital stress on cancer cells, initiating cancer-cell necrosis. Sui’s group coupled two CPT molecules with an oligomer chain of ethylene glycol (OEG), synthesizing amphiphilic phospholipid-mimicking prodrugs [47]. These obtained prodrugs could form stable liposome-like nanocapsules with high drug-loading efficacy, which achieved higher antitumor activity in vitro and in vivo.

To further endow prodrug nanoparticles with active targeting capacity, some peptides or other molecules were integrated into the prodrugs [48]. These prodrug nanoparticles could specifically recognize receptors on the surface of the cancer-cell membrane. Arginine-glycine-aspartic acid (RGD) peptide is a typical targeting peptide to recognize the integrin αvβ3 receptor [49]. Zhang’s group conjugated CPT and a hydrophobic tail to the end of the Arg-Gly-Asp-Ser (RGDS) peptide, which could self-assemble into fibrillary nanoarchitectures and effectively target integrin-overexpressed cancer cells. Based on their results, this nanomedicine could significantly suppress tumor growth via RGD-mediated specific targeting [50]. Yan’s group utilized lactose (Lac) as the targeting ligand to modify the DOX. The Lac-DOX conjugate could self-assemble into nanoparticles in aqueous solution [14]. Moreover, both in vitro and in vivo evaluation demonstrated that the Lac-DOX nanomedicine showed improved antitumor activity and alleviative side effects. 

### 2.3. Drug–Drug Conjugate Nanoparticles

Drug–drug conjugates are a particular kind of amphiphilic prodrug, deserving of a separate discussion. Different from polymer-drug or peptide-drug nanoparticles, amphiphilic drug–drug conjugates are composed of hydrophobic and hydrophilic antitumor drugs. They can also self-assemble into nanoparticles without additional stabilizers, yielding relatively stable drug-delivery systems. In recent years, Yan’s group prepared a series of amphiphilic drug–drug pairs by conjugating water-soluble antitumor drugs to poor water-soluble drugs via a hydrolysable ester linker such as irinotecan-chlorambucil (Ir-Cb) [51], irinotecan-doxorubicin (Ir-DOX) [52], floxuridine-bendamustine (FdU-BdM) [53] and floxuridine-CPT (FdU-CPT) [54]. From there, Ir-DOX conjugate nanoparticles were capable of overcoming multidrug resistance (MDR) on breast cancer. Due to aggregation-caused quenching (ACQ) of both Ir and DOX in these nanoparticles, the blue fluorescence of Ir and red fluorescence of DOX were quenched significantly. However, after the Ir-DOX linkage broke and Ir and DOX were released, dual-color fluorescence was recovered, which could be used to real-time track two drugs during cancer therapy. To endow drug–drug conjugate nanoparticles with environment-sensitive release behavior, some sensitive linkers, represented by an ester bond and disulfide bond, were adopted into drug–drug conjugates. For example, Chen’s group utilized redox-responsive disulfide linkers to connect evans blue (EB) with CPT, which could self-assemble into nanoformulation in water [55]. Meanwhile, the redox-responsive drug-release manner in cancer cells was also accordingly proved. This cargo-free nanomedicine showed 130-fold longer blood circulation and more efficient tumor accumulation than free drugs, which were beneficial to achieve excellent therapeutic efficacy.

Instead of using covalent bonds to form drug–drug conjugates, Zhu’s group employed noncovalent molecular interaction to link hydrophobic (quinazoline-based folate analogue, RT) and hydrophilic drugs (purine nucleoside analog, CA) together [56]. Through computer simulation and transmission electron microscope (TEM) studies, it was demonstrated that the nanomedicine formed by the resultant amphiphilic drug–drug conjugates exhibited stable nanostructures with higher drug loading. Additionally, these nanodrugs were able to simultaneously inhibit ribonucleotide reductase, DNA polymerase and thymidylate synthase through the synergetic effect of CA and RT both in vitro and in vivo.

**Figure 2:**
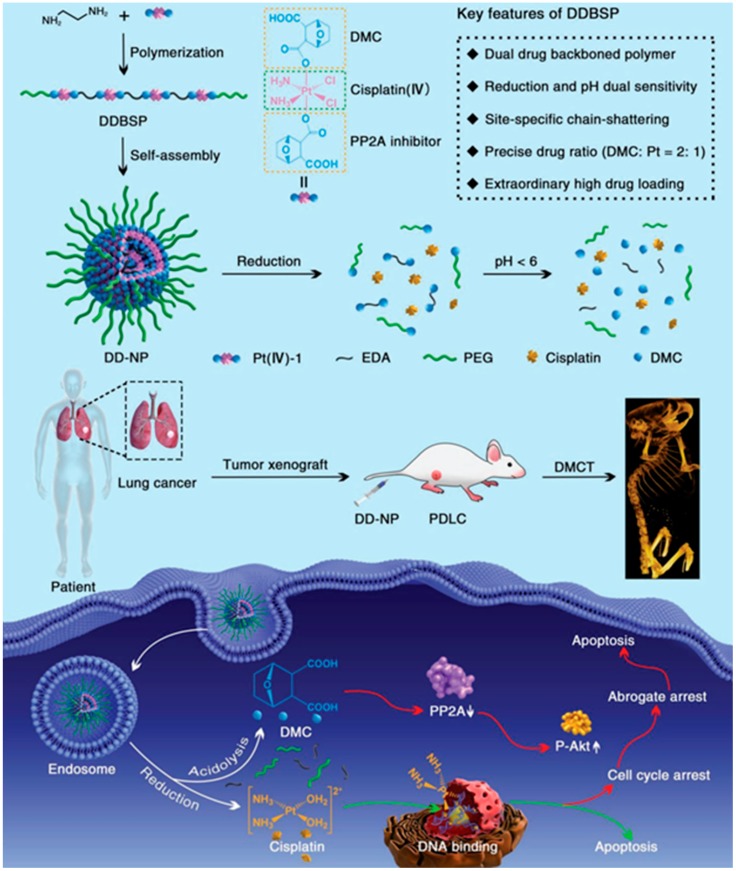
Schematic illustration of a polymeric drug consisting of cisplatin and demethylcantharidin for computer-tomography (CT) imaging and cancer therapy [57].

Moreover, anticancer drugs could be used as a monomer to synthesize polymeric drugs. These polymeric drugs could self-assemble into nanoparticles and present impressive pharmacological activity. For example, Zhou’s group selected the antitumor drugs, including cisplatin (Pt(IV)) and demethylcantharidin (DMC, protein phosphatase 2A (PP2A) inhibitor), as monomers for polymerization with ethylenediamine (EDA) via *N*-hydroxysuccinimide (NHS)/1-ethyl-3-(3-dimethylaminopropyl) carbodiimide hydrochloride (EDC), eventually forming the polymeric drugs (Figure 2) [57]. Without inducing any additional carriers, the nanomedicine prepared by the resultant polymeric drugs possessed several excellent advantages: (1) The ratio of Pt(IV) and DMC in polymeric drugs can be precisely controlled; (2) due to the existence of a heavy metal (Pt) in polymeric drugs, this nanomedicine could also be utilized as a computer tomography (CT) imaging agent to monitor the tumor’s status during the therapy; (3) this nanomedicine can effectively suppress the tumor burden on a patient-derived lung-cancer (PDLC) model. 

### 2.4. ADCs

In recent decades, ADCs have also been recognized as an attractive solution to overcome challenges in cancer therapy. By exploiting the specific binding ability of monoclonal antibodies (mAb) to cell-surface target antigens, ADCs could selectively deliver cytotoxic drugs to tumor cells. All three components of ADCs, mAb, cytotoxin and the chemical linker that connects them together, are crucial for developing highly efficient ADCs, making ADCs an important type of cargo-free nanomedicine for cancer therapy. The first action of ADCs after being administered intravenously is to recognize the specific antigen on the surface of target cells by its antibody component. Then, the ADC/antigen complex could be internalized by cancer cells through receptor-mediated endocytosis. After transporting the payloads to endosomes and lysosomes, the complex is degraded and the drugs are further released into the cytoplasm to induce cell death with various mechanisms, mainly depending on the type of the toxic drugs. Currently, several ADCs have been approved by the FDA for clinical cancer treatment and over 50 ADCs are in the process of clinical trials [58,59]. In 2000, Gemtuzumab ozogamicin (GO), as the first FDA-approved ADC and the first-generation ADC, was used for monotherapy for patients with acute myeloid leukemia (AML), who were not suitable for conventional chemotherapy. This ADC contains a humanized IgG4 mAb against CD33, a surface antigen that presents in 85–90% of AML patients, which is conjugated to calicheamicin through a cleavable hydrazone linker. The outcomes of GO in the initial single-agent Phase II trials were positive, with a remission rate of 30% [60]. Nevertheless, the required post approval study demonstrated no improvement in survival rate and even higher fatal toxicity in patients treated with GO and chemotherapy compared with patients treated with chemotherapy alone, resulting in the voluntary withdrawal of GO in 2010 [61]. 

In 2013, one of the second-generation ADCs, ado-trastuzumab emtansine (T-DM1), was approved by the FDA for the treatment of non-hematological malignancies. In T-DM1, a non-cleavable linker connects the well-known antibody trastuzumab (humanized IgG1 anti-HER-2 antibody) and drug maytansinoid DM1. Trastuzumab could not only play the role of active targeting ligand for binding to the HER-2/neu receptor on target cancer cells but also prevent homodimerization or heterodimerization (Her2/Her3) of receptors, finally inhibiting the activation of MAPK and PI3K/AKT cellular signaling pathways to stop the growth of cancer cells. Meanwhile, DM1 are internalized into cancer cells followed by binding to tubulin to induce cell death [62,63]. However, there is a black-box warning of serious side effects, such as hepatotoxicity, embryofetal and cardiac toxicity for T-DM1, creating a need for third-generation ADCs [63]. 

Vadastuximab talirine, also known as SGN-CD33A, is representative of third-generation ADCs, containing a novel synthetic pyrrolobenzodiazepine (PBD) dimer, which is structurally similar to anthramycin. A humanized anti-CD33 IgG1 antibody is coupled to PBD through a maleimidocaproyl valine-alanine dipeptide linker. Vadastuximab talirine showed robust activity in a series of AML animal models, including those in which GO had minimal effect [64,65]. Moreover, CD33-directed delivery of PBD dimers could overcome transporter-mediated multidrug resistance. Currently, vadastuximab talirine, in combination with azacitidine or decitabine, is undergoing evaluation via a Phase III trial in older patients with newly diagnosed AML. 

Apart from ADCs under clinical trials, there is a large number of ADCs in the research phase that are also showing potential for improving cancer therapy [66,67,68]. Through the exquisite design in the components of mAb, linker and cytotoxic drugs, these ADCs could be considered in the development of potent antitumor therapeutic agents in the future.

## 3. Representative Applications of Cargo-Free Nanomedicine

Due to tumor heterogeneity [69], single-drug treatment is usually not adequate to eliminate cancer. Additionally, MDR acquired after repeated treatment with single chemotherapeutics could enhance the possibility of tumor metastasis or recurrence after traditional treatments [70]. Thus, combinational treatment with multiple chemotherapeutics or therapeutic modalities is considered a more promising way of cancer therapy. As described above, cargo-free nanomedicine has the potential advantage of incorporating multiple therapeutics into a single nanoplatform in an easy manner, which is beneficial for achieving combination-cancer therapy through a synergetic effect. In the following section, we present some representative applications of cargo-free nanomedicine for combined cancer therapy. 

### 3.1. Chemo–Chemotherapeutics Combination Therapy

The basic principle of chemo–chemotherapy combination therapy is to use drugs with different therapeutic mechanisms and deliver multiple drugs at the maximum tolerated dose. There is a growing awareness that synergistic cytotoxicity could be achieved both in vitro and in vivo with the appropriate combination of drugs. However, the administration of a drug combination through the conventional free “cocktail” method has shown unsatisfactory therapeutic outcomes in vivo due to the different distribution and metabolism of individual drugs. Additionally, a drug combination may result in unexpected systemic toxicity. Therefore, it is necessary to employ an effective system to achieve chemo–chemotherapeutics combination therapy. Due to excellent features, cargo-free nanomedicine is one candidate to achieve this goal. For example, Wu’s group prepared a dual-drug nanohybrid that consisted of 10-HCPT nanocrystals integrated with an exterior layer of the methotrexate (MTX)-chitosan prodrug [71]. Since MTX could simultaneously be used as a chemotherapeutic agent for killing cancer cells and a ligand for tumor targeting, this HCPT- and MTX-based dual-drug nanoformulation possessed enhanced cellular internalization and antitumor capacity in Hela cells when compared to either individual drug or their physical mixture. Similarly, Zhang’s group employed three poor water-soluble drugs, namely, HCPT, MTX and paclitaxel (PTX), to fabricate cargo-free multidrug nanocrystals (MDNCs) for combination chemotherapy [72]. Through the synergetic effect of these three drugs, this MDNC could effectively overcome MDR to achieve significantly superior anticancer efficacy. MDNCs could also be used as an imaging agent by loading some imaging probes, which render the multidrug nanorods as an all-in-one processing system for cancer diagnosis and treatment. 

**Figure 3:**
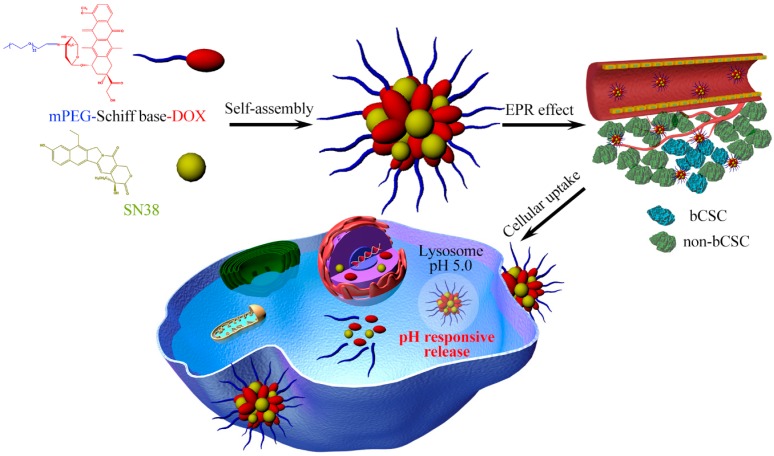
Schematic illustration of pH-responsive cargo-free nanomedicine with improved cellular uptake and controlled drug release for the elimination of bCSCs (breast cancer stem cell) and non-bCSC [76].

In recent years, it has been found that there exists a very small population of cancer cells (cancer stem cells, CSCs) in tumors that possess tumor-growth, metastasis and recurrence abilities [73,74,75]. Thus, our group designed and prepared a pH-responsive cargo-free nanomedicine consisting of pH-responsive prodrug (PEG-C = N-DOX) and 7-ethyl-10-hydroxyl camptothecin (SN38) through their self-assembling. The synergistic effect of the two drugs could simultaneously eradicate breast-cancer cells and cancer stem cells (Figure 3) [76]. In this system, because of the pH-responsive imine linker between DOX and PEG, this cargo-free nanomedicine could achieve pH-controlled release and avoid unexpected drug release during circulation. More importantly, because topoisomerases in CSCs play a key role in facilitating transcription rate, enhancing the expression of antiapoptotic proteins and repairing DNA, the inhibition of both TOP I and TOP II using DOX and SN38 can effectively eliminate non-CSCs and CSCs, improving in vivo therapy effect with fewer adverse effects [77]. 

### 3.2. Chemo–Photosensitizer Combination Therapy

#### 3.2.1. Chemo–Photodynamic Combination Therapy

Photodynamic therapy (PDT) is increasingly recognized as an alternative to treating various cancers in clinical trials due to its simplicity, efficiency and fewer side effects [78,79,80]. PDT uses a light source at appropriate wavelengths to activate tumor local photosensitizers, producing highly toxic reactive oxygen species (ROS), such as singlet oxygen (^1^O_2_) and free radicals. Then, ROS can directly induce cytotoxic effects in tumor cells, damage to the tumor vasculature and a strong inflammatory response that can lead to systemic immunity [78]. The combination of chemotherapy and PDT has been widely studied in recent years, the results of which found that chemo–photodynamic combination therapy can significantly maximize therapeutic efficiency against cancer [81]. As a multifunctional delivery platform for cancer therapy, cargo-free nanomedicine containing several chemotherapeutics and photosensitizers were employed for combined chemo–PDT.

Chlorin e6 (Ce6) is a second-generation photosensitizer, synthesized from chlorophyll. Upon light irradiation by light in the red spectrum (655 nm), the activated Ce6 could efficiently generate ^1^O_2_ [82]. Moreover, there exist obvious clinical benefits achieved by the Ce6-mediated PDT on various cancer treatments, such as bladder cancer, melanoma and nasopharyngeal cancer. Therefore, various kinds of cargo-free nanomedicines composed of Ce6 have been developed for chemo–photodynamic combination therapy. For example, Liang’s group employed a green and simple reprecipitation method to form stable carrier-free pure nanodrugs (PNDs) consisting of Ce6 and HCPT [82]. Based on their results, it has been found that the ROS-generation efficiency of Ce6 and the cytotoxicity of HCPT were not influenced during the co-self-assembly process. In addition, HCPT/Ce6 PNDs were able to effectively accumulate in tumor tissue due to their nanoscale features. The results of in vitro and in vivo antitumor studies exhibited that chemo–photodynamic combination therapy possessed obviously enhanced anticancer efficacy when compared to single chemotherapy or PDT. Tumor growth was also remarkably suppressed when treated with HCPT/Ce6 PNDs upon light irradiation. It is well known that Ce6 and some chemotherapeutic agents that exist in the aromatic structure can self-assemble into nanoformulation while excluding unnecessary materials. 

**Figure 4:**
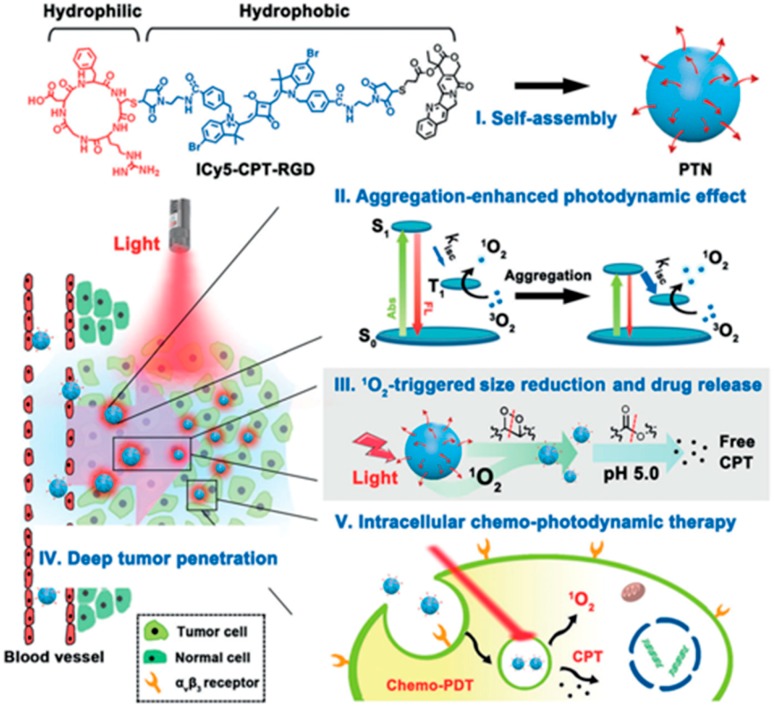
Schematic illustration of cargo-free nanomedicine formed by targeted drug–drug conjugates (ICy5-CPT-RGD) for chemo–photodynamic therapy [80].

Yoon’s group used a hydrophilic photosensitizer (zinc(II) phthalocyanine tetrasubstituted with 6,8-disulfonate-2-naphthyloxy groups (PcS)) and mitoxantrone (MA) as the host and guest molecules for supramolecular assembly [83]. The resultant nanomedicine displayed enhanced PDT by increasing intratumoral blood flow and relieving tumor hypoxia. Meanwhile, the concomitant chemotherapeutic effect due to the formation of free MA proved to eliminate deeply located tumor cells out of the range of PDT. Similarly, Yin’s group also developed a targeted phototheranostic nanodrug (PTN) by covalently conjugating CPT (chemotherapeutic agents) with cyclic RGD (targeting peptides) and pentamethine indocyanine (ICy5, photosensitizers) for self-assembly (Figure 4) [80]. Due ICy5 aggregation, ^1^O_2_ generation efficiency was significantly enhanced upon red-light irradiation. Meanwhile, particle size decreased from 90 nm to 10 nm, which was beneficial for deeper tumor penetration as well as CPT release. Thus, this targeted PTN obtained 77.5% tumor-inhibition efficiency through chemo–photodynamic combination therapy. In contrast, the value was only 33.1% and 20% for the single PDT or chemotherapy, respectively. It was indicated that this multifunctional nanodrug would be a promising platform to improve antitumor performance. 

#### 3.2.2. Chemo–Photothermal Combination Therapy

In recent years, photothermal therapy (PTT), an extension of photodynamic therapy, has attracted great attention for cancer therapy. Photosensitizers in an excited state activated by near-infrared (NIR) radiation can release energy via non-radiative vibrational relaxation to induce local hyperthermia and cell death, which is the basal principle for organic photosensitizer-mediated PTT. There are numerous advantages for this therapeutic modality, such as increased preservation of surrounding tissue, minimized invasiveness, short recovery time and reduced complication rates [84]. More importantly, it was found that the local hyperthermia effect can assist in the cellular uptake of chemotherapeutics and facilitate the therapeutic efficiency of chemotherapeutic agents both in vitro and in vivo [85]. Thus, a growing number of nanomedicines in combination of chemotherapy and PTT have been fabricated and used to suppress tumor growth through their synergetic effect.

During PTT, photothermal agents play an important role in improving the chemo–photothermal combination therapeutic effect. Various inorganic and organic photothermal agents have been prepared. For inorganic photothermal agents, different carbon nanoformulations [86], gold nanostructures [87], copper sulfide nanoparticles [88], palladium nanosheets [89] and a few others were included [90]. Although these inorganic photothermal agents can effectively transform absorbed NIR optical energy into heat, they still show some disadvantages, such as nonbiodegradability and long-term retention in the body. Due to the potential toxicity caused by inorganic PTT agents to the human body, their further application in the clinic has been limited. In contrast, organic photothermal agents not only possess high NIR absorption and photothermal conversion efficiency but also good biodegradability and structural modifiability. Therefore, organic photothermal agents are usually combined with chemotherapeutics and exploited to construct cargo-free nanomedicine for chemo–photothermal combination therapy. 

Indocyanine green (ICG) is a cyanine dye (fluorescent dye) that has been approved by the FDA for medical diagnostics in clinics [91]. It was demonstrated that ICG has better capacity of transforming NIR light energy into heat to induce hyperthermia [92]. Thus, it has also been applied for PTT and photoacoustic (PA) imaging for cancer therapy and diagnosis simultaneously. Hou’s group reported a chemotherapeutic drug-photothermal agent, which was composed of ICG and another FDA-approved antitumor drug, epirubicin (EPI), by co-self-assembling into nanoformulation through collaborative interactions (π–π stacking and electrostatic and hydrophobic interactions) in the absence of a molecular precursor and excipient [93]. Similar to other cargo-free nanomedicine, these ICG-EPI nanodrugs has high drug loading (~92%) and excellent stability in physiological conditions. Moreover, due to their preferable photothermal conversion efficacy, these nanodrugs exhibited enhanced synergistic chemo–PTT efficiency and inappreciable toxicity compared to free ICG or EPI. More importantly, as described above, ICG also was a near-infrared fluorescent (NIRF) and PA imaging agent; ICG-EPI nanodrugs also showed high spatial resolution and deep penetration via in vivo NIRF/PA dual-modal imaging. Thus, a multifunctional nanotheranostic platform was able to obtain the targeting multinadal imaging-guided cancer therapy and real-time self-monitoring of drug release. Similarly, Shao’s group prepared a targeted nanodrug formed by the co-self-assembling of ursolic acid (UA) as antitumor drug, ICG as PTT and PDT agent and lactobionic acid (LA) as targeting ligand [94]. This nanodrug was then successfully used for cancer imaging and chemo–photo combination therapy on asialoglycoprotein receptor (ASGPR)-overexpressing human hepatocellular carcinoma (HepG2). It is worth mentioning that this multifunctional nanoparticle can simultaneously combine chemotherapy, PTT and PDT to treat cancer under the guidance of NIR-fluorescence imaging, which is essential for a precise and synergistic antitumor effect. 

**Figure 5:**
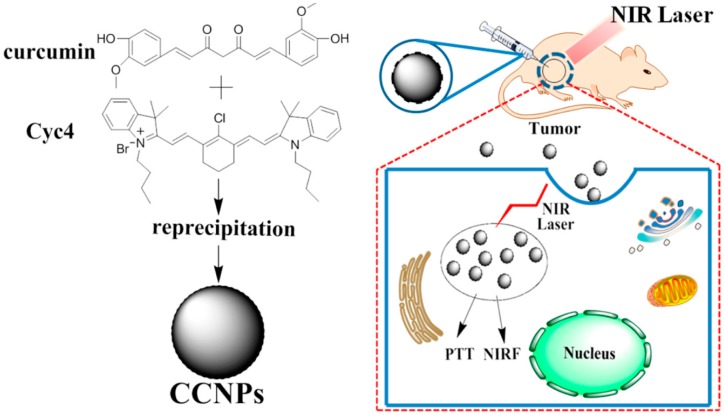
Schematic illustration of self-assembly of CUR and Cyc4 for chemo–photothermal therapy [95].

Additionally, Jing’s group developed cyanine–curcumin self-assembling nanoformulations (CCNPs) through a single-step reprecipitation method (Figure 5) [95]. The resultant nanomedicine not only showed in vivo photothermal tumor ablation under NIR laser irradiation but also had excellent NIR-imaging ability, indicating that this multifunctional cargo-free nanomedicine would be a promising strategy for drug delivery and cancer treatment. 

### 3.3. Other Applications

#### 3.3.1. Immunotherapy

As an effective way for cancer therapy, cancer immunotherapy has had much focus in recent years, as it can eliminate both metastatic cancer cells and primary cancer cells [96,97,98]. In addition, with the development of nanotechnology, a growing number of nanoparticles were employed for cancer immunotherapy, including vaccines, immune checkpoint blockade therapy and adoptive T-cell transfer [99,100]. Liu’s group prepared cancer-cell membrane-modified adjuvant nanoparticles as a vaccine for cancer therapy [101]; You’s group developed hollow gold nanoshell (HAuNS)-loaded PLGA nanoparticles to encapsulate antiprogrammed death 1 (PD-1) peptides, which achieved a preferable anticancer effect through the combination of PTT and immunotherapy [102]. 

Cargo-free nanomedicines can also be applied for immunotherapy. Ammonio methacrylate copolymers (AMC), the components of controlled-release oral formulations, have been well studied in the pharmaceutical field [103]. Lamprecht’s group prepared cargo-free particles of AMC in small (130 nm) and large (500 nm) sizes for immunotherapy [103]. Since immunomodulation can be regulated by different factors of particles, including size, surface charges, composition and morphology, AMC nanoparticles with different size can activate varying immune responses. Based on their results, the level of IL-6, TNF-α, G-CSF and RANTES (CCL5) on immune cells treated with AMC nanoparticles increased with the size of nanoparticles. Moreover, the secretion of other cytokines, like IL-12p40 and IFN-γ, could be facilitated upon cellular exposure to quaternary ammonium groups, all of which induced the apoptosis of cancer cells and immunological memory.

**Figure 6:**
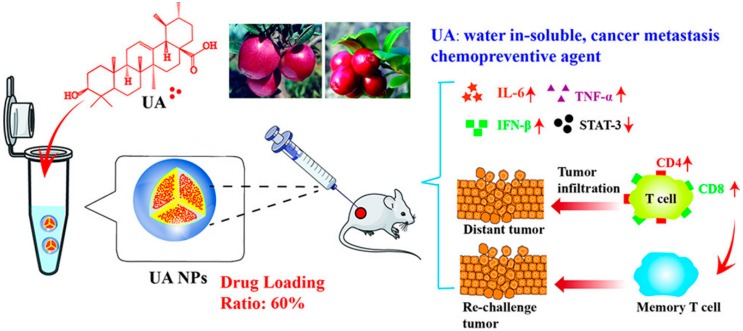
Cargo-free self-delivery system developed by natural anticancer drug ursolic acid for immunotherapy [104].

As shown in Figure 6, Shao’s group also prepared a carrier-free nanodrug by self-assembly of a pure drug (ursolic acid, UA), which is a food plant-derived natural product possessing good capacity for cancer therapy. As shown in Figure 6, this cargo-free nanomedicine with a diameter of 150 nm could not only induce antiproliferative activity but also inhibit the expression of COX2/VEGFR2/VEGFA and facilitate the immunostimulatory activity of IL-6, IFN-β and TNF-α. Thus, it significantly caused the apoptosis of A549 cells in vitro and suppressed tumor growth in vivo. Additionally, compared to the free drug, this UA carrier-free nanodrug further improved immune function and anticancer efficacy [104]. 

#### 3.3.2. Real-Time Self-Monitoring of Drug Release

**Figure 7:**
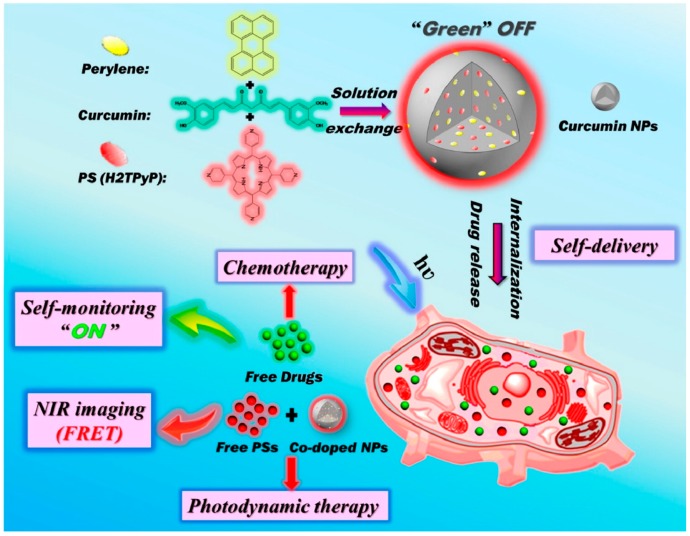
Schematic illustration of a self-monitored drug-delivery system composed of curcumin (CUR), perylene and 5,10,15,20-tetro (4-pyridyl) porphyrin (H2TPyP) for self-monitoring drug release, NIR imaging and cancer therapy [106].

Through an elaborate design by researchers, cargo-free nanomedicines have also been used to real-time self-monitor drug release or track cancer cells as an extension for the application of these nanoparticles for cancer diagnosis. Liang’s group employed anticancer drug curcumin (CUR) to construct cargo-free medicine with the reprecipitation method, followed by surface modification with poly(maleic anhydride-alt-1-octadecene)-polyethylene glycol (C18PMH-PEG) through hydrophobic interactions to facilitate further in vivo applications [105]. The green fluorescence of CUR was quenched significantly in the nanomedicine due to serious aggregation and fluorescence would not be restored unless CUR could be released from the nanoparticles. Through their studies by confocal laser-scanning microscope and flow cytometry, the green fluorescence of CUR could not be detected in cancer cells at the beginning of this nanomedicine treatment. However, after longer incubation, CUR’s green-fluorescence intensity was gradually enhanced, indicating that this cargo-free nanomedicine exhibited time-dependent cellular release capacity, which could be monitored by the nanomedicines themselves. Similarly, Lee’s group developed another self-monitored drug-delivery system, which was composed of CUR, perylene and 5,10,15,20-tetro (4-pyridyl) porphyrin (H2TPyP) (Figure 7) [106]. In this system, CUR could also inhibit cancer-cell growth and self-monitor release by observing their green “ON–OFF” fluorescence. It is worth mentioning that ^1^O_2_ generation efficiency could be enhanced via Förster Resonance Energy Transfer (FRET) from perylene under NIR laser irradiation. Therefore, this self-assembled nanomedicine was regarded as a real-time self-monitored and self-delivered drug-delivery system, capable of multiple-fluorescent imaging and chemo–photodynamic therapy.

Zhang’s group developed surface-functionalized small-molecule organic-dye nanoparticles for cell imaging [107]. Based on their design, poly(maleic anhydride-alt-1-octadecene)-polyethylene glycol (C18PMH-PEG) was selected as the surfactant to surface-modify the preformulated pure organic-dye (2-tert-butyl-9,10-di(naphthalen-2-yl)anthracene, TBADN) nanoparticles. Due to the absence of additional inert materials as carriers, the dye-loading capacity of the resulting TBADN NPs was much higher than those reported carrier-based nanoparticles, leading to significantly enhanced photoluminescence intensity, which was even higher than quantum dots (CdSe/ZnS) under the same conditions. Especially when this pure dye NPs is modified with the targeting ligands (Folic acid), it could be an efficient targeted fluorescence probe for tracking cancer cells.

## 4. Challenges and Future Perspectives of Cargo-Free Nanomedicine

Although cargo-free nanomedicine can avoid carrier toxicity, effectively deliver antitumor agents to tumor sites and exhibit excellent therapeutic efficiency, it still has a long way to go to be further developed for clinical applications. To further improve the performance of cargo-free nanomedicine, the following two challenges should be carefully considered. 

The first challenge is the enhancement of the EPR effect. It is well known that a large number of nanomedicines have been developed for cancer therapy, the effective performance of which was largely depended on the EPR effect. Unfortunately, few of them have accomplished clinical transformation, which is not only attributed to their complicated fabrication processes but also the poor EPR effect they have on humans [11]. In mice, the tumor grows rapidly and blood vessels in tumors tend be much leakier, achieving an effective EFR effect. However, not all tumor vessels are leaky in human tumors; the heterogeneous distribution of pore sizes in human tumors usually compromises the intratumoral accumulation of nanomedicine, resulting in inadequate therapeutic efficacy. To solve this problem, it is necessary to select the most appropriate tumors for cargo-free nanomedicines to exert therapeutic action. In clinics, several kinds of tumors, such as head and neck tumors and Kaposi sarcoma, have been proven to possess an excellent EPR effect and they could be selected as the first series of treatment objects [11]. Using auxiliary approaches to enhance the EPR effect in human tumors could also be another promising way for successful cancer treatment with cargo-free nanomedicine. For example, Jaffray’s group found that heat and radiation could obviously facilitate the EPR effect and delivery efficiency of nanomedicine by adjusting human intratumoral-fluid dynamics [108]. Thus, combination of radiation or heat with cargo-free nanomedicine may also be a potential way to improve the therapeutic effect of nanomedicine on human cancer. 

The second challenge for cargo-free nanomedicine is to precisely control drug ratios in multidrug nanoparticles. It is demonstrated that combination therapy using more than two kinds of drugs could achieve a better therapeutic effect. Moreover, to maximize efficiency, it is necessary to optimize the ratios of different drugs in nanomedicine or results backfire due to additional side effects of more chemotherapeutics. However, for most prodrugs or drug–drug conjugates, it is hard to precisely control their ratios. Thus, it is urgent to design and develop more reasonable ways to construct cargo-free nanomedicine, like polymeric drugs. 

Through the discussion of their advantages, applications and challenges, it is believed that cargo-free nanomedicines are an effective and promising drug-delivery system for cancer treatment. After considerable progress in clinical applications, it is also expected that multifunctional cargo-free nanomedicine would be a milestone in the near future for cancer therapy using nanotechnology.

## Figures and Tables

**Table 1:** Clinical-stage nanomedicines for cancer treatment [7].

Trade Name	Nanotechnology Platform	Cancer Type	Status
Doxil	Liposome	Ovarian cancer, HIV-related Kaposi sarcoma and multiple myeloma	Approved by the U.S. Food and Drug Administration (FDA)
Genexol-PM	Micelle	Breast cancer and non-small-cell lung cancer	Approved in Korea
Abraxane	Albumin NP	Lung, breast and pancreatic cancer	Approved by FDA
CRLX-101	Polymeric NP	Metastatic renal-cell carcinoma, non-small-cell lung cancer and recurrent tubal, ovarian, or peritoneal cancer	Phase II
CYT-6091	Colloidal gold NP	Advanced solid tumors	Phase I
Kadcyla	Antibody-drug conjugate	HER-2 positive breast cancer	Approved by FDA
